# Digital Versus Conventional Teaching of Surgical Pathology: A Comparative Study

**DOI:** 10.7759/cureus.45747

**Published:** 2023-09-22

**Authors:** Pushpak Chaudhari, Shilpa Gupta, Shanu Srivastav, Vivek Sanker, Gnana Deepthi Medarametla, Akash Pandey, Yash Agarwal

**Affiliations:** 1 Surgery, Terna Speciality Hospital and Research Centre, Mumbai, IND; 2 Pathology, Terna Medical College, Mumbai, IND; 3 General Surgery, Noorul Islam Institute of Medical Science (NIMS), Trivandrum, IND; 4 Internal Medicine, Amma Multispeciality Hospital, Thorur, IND; 5 Internal Medicine, Dr. Rajendra Prasad Government Medical College, Tanda, IND; 6 Medicine, West Bengal University of Health Sciences, Kolkata, IND

**Keywords:** microscopy slides, biopsy, pathology, conventional, digital

## Abstract

Objective: To compare the digital method and the conventional method of teaching surgical pathology to medical students.

Methods: A prospective case-control study was conducted on second-year students during the period of August 20, 2022, through January 15, 2023. Students, divided into two groups of 45 each, were taught surgical pathology via both conventional and digital methods. Four specimens and four slides were taught in total to the same set of students. A pre-test and a post-test were used to evaluate students’ performance and the impact of the teaching method. The answers were analyzed using a paired t-test. In the end, students’ responses were obtained regarding their views on a better method of teaching on a Likert scale.

Results: To study gross pathology, 50.7% of students were in favor of the digital method, and 21% were not in favor. For the microscopic examination of tissues, 56.92% of students were in favor of the digital method, and 15% were not in favor. There was a significant increase in post-test scores (12.54-9.79 = 2.75, p=0.007) when digital methods for teaching surgical pathology were applied.

Conclusion: The Likert scale demonstrated that the digital method of teaching surgical pathology not only improved student performance but also resulted in a better understanding of the subject.

## Introduction

Significant technological advancements in recent years have been used in almost every aspect of human life, including education. During the last decade, pathology has benefited from the rapid progress of image-digitizing technology [1]. Histopathological analysis of diseased tissues forms part of the foundation for pathological studies, which is a highly visual field. Microscopic and gross examination of pathological specimens can provide insights into the underlying disease, which can help medical students better visualize histological changes and abnormalities in tissues and organs, which is essential for understanding the disease process [2,3]. The employment of these new technologies in teaching has improved many dental and medical disciplines.

Microscopes have been employed in the field of pathology since advances in lens production at the turn of the 19th century, and there has been little advancement afterward. Since the 1990s, multimedia has gradually replaced these traditional teaching methods [4]. Presently, several educational systems use advanced technology to cope with the demands of students and academic faculties. Countless studies have shown that an amalgamation of conventional and electronic teaching methods can improve learning outcomes [5].

In recent years, digital microscopy has gained popularity as a teaching tool in pathology for medical students. In comparison to conventional methods, digital microscopy offers numerous advantages, such as improved accessibility, ease of sharing, and the ability to zoom in and out, among others. The COVID-19 pandemic has forced traditional medical teaching to be restructured and delivered online [4]. Many educators have adapted their approaches to accommodate remote learning and reduced access to laboratory facilities. Virtual microscopy and other online tools are effective for teaching pathology during the pandemic and have been proven in multiple studies to enhance students' learning processes [6], which may also have long-term benefits for the field.

A student's decision to pursue pathology as a career path may be positively influenced by exposure to digital microscopy and other powerful teaching resources in the field [7]. The idea of virtual slides, which completely replicate a conventional microscope and glass slides, makes it feasible to analyze histopathological sections at various degrees of magnification without influencing the quality. Digitalization has infiltrated every aspect of life. Emerging new technologies have an impact on our lives and change the way we do our daily work, raising our performance to a new height. The technology used in education has allowed educators to implement new theories to enhance the teaching and learning process. This shift from “traditional learning” has led to a model in which learning can take place outside the classroom, facilitating different learner attributes such as visual, verbal, aural, and solitary learning within the “blended learning” approach [8].

The integration of conventional and technology-based methodologies in the study of histopathology has been effective because of the advancements in technology. Digital teaching can provide greater accessibility to medical students who may not have access to traditional classroom-based learning due to geographic or time constraints. Students can learn at their own pace by accessing course materials and lectures using a computer or mobile device, as there will be greater flexibility in terms of scheduling and course delivery. Instructors can tailor the content to meet individual learning needs, allowing for more personalized and customized learning experiences [9-11]. This prompted numerous medical schools worldwide to adopt new technology to teach pathology and histopathology in their regular undergraduate courses [12].

The purpose of the current publication is to discuss our group's experience moving from conventional glass slide microscopy to digital pathology, emphasizing the challenges and advantages that students, lecturers, and the institution faced.

## Materials and methods

Study protocol

The study was approved by the institutional ethical committee (TMCHRC/Surg/2022/IEC Protocol-17/64) to conduct a comparative study. After explaining the aim and method of the study, consent was obtained from each participant involved in the study. A total sample size of 90 students was chosen by using systematic random sampling.

Study participants

The assessment was conducted on two-year medical students at Terna Medical College, Nerul, India. The 90 students were divided into two groups, with 45 students in each group. Both groups were taught separately via conventional and digital methods. In the conventional teaching method, students were taught on two fresh specimens preserved in formalin and two glass slides. In the digital method, however, students were taught using images of two slides and two gross specimens. Four slides and four gross specimens were taught throughout the study (Table 1).

**Table 1:** Slides and gross specimens taught throughout the study

	Conventional	Digital
Specimens	Tubercular lymph node and fibroadenoma	Appendicitis and leiomyoma
Slides	Tubercular lymph node and fibroadenoma	Appendicitis and leiomyoma

The specimens and slides were selected based on the most common cases in the region and the most frequently asked questions in the examination.

Pre-test and Post-test

The first group was given a pre-test, then taught two specimens and two slides conventionally, followed by a post-test. They were then given another pre-test, taught two different specimens and two different slides digitally, followed by a post-test. The same procedure was repeated for the second group. A pre-test and a post-test were used to evaluate students’ performance and the impact of the teaching method. The answers were analyzed using a paired t-test. In the end, students’ responses were obtained regarding their views on a better method of teaching on a Likert scale. The questionnaire was provided to students in the form of a Google form. Face validity and content validity were considered in the development of the questions. A pre-test was administered, then the histopathology slides and specimens were traditionally taught to the students using histopathological glass slides and fresh specimens preserved in formalin, and later a post-test was administered. The same procedure was used in the digital teaching method, in which students were taught using images of slides and gross specimens on a projector screen. Each correctly answered question was scored 1 point; there was no negative scoring. A total of five questions were asked for each specimen and slide. The questions were designed considering the overall learning objectives, such as why, how, what, etc.

Students’ feedback questionnaire

At the end, a questionnaire (5-point Likert scale having the following variables: strongly agree, agree, neutral, disagree, and strongly disagree) was completed on students' views of the different teaching methods to assess the superiority of the digital method compared to the conventional method. Two feedbacks were obtained: one on microscopic pathology in terms of cell morphology, nuclear morphology, visualization of cell arrangements, staining of cells, and overall pathology in terms of microscopy, and the other on gross pathology in terms of various aspects such as 3D shape and size, relationship of the specimen within the organ, characteristics of the specimen, and overall pathology in terms of gross specimen.

Statistical analysis

A paired t-test was performed to analyze the difference between the pre-test and post-test results. SPSS version 26 software (IBM Corp., Armonk, NY) was used for statistical analyses. A 5-point Likert scale was used to assess student opinion of the new method (digital method).

## Results

Ninety students (two groups) studied two specimens (tuberculous lymph nodes and fibroadenoma) and two slides (tuberculous lymph nodes and fibroadenoma) by the conventional method using fresh, formalin-preserved specimens and slides. The minimum score was 3 on both the pre-test and post-test, and the maximum score was 18 points in the pre-test and 17 points in the post-test. The average score on the pre-test was 10.43, while in the post-test it was 11.24 (Table 2).

**Table 2:** Descriptive analysis of the conventional method

	N	Minimum	Maximum	Mean	Std. deviation
Conventional pre-test scores	90	3	18	10.43	3.568
Conventional post-test scores	90	3	17	11.24	3.783
Valid N (list wise)	90				

The same two groups of 90 students were taught using images of slides and microscopic specimens on a projector with two other slides and specimens (Appendicitis and Leiomyoma). The minimum score on the pre-test was 5 and on the post-test was 4. The maximum score was 15 on the pre-test and 20 on the post-test. The average score was 9.79 on the pre-test and 12.54 on the post-test (Table 3).

**Table 3:** Descriptive analysis of the digital method

	N	Minimum	Maximum	Mean	Std. Deviation
Digital pre-test scores	90	5	15	9.79	2.714
Digital post-test scores	90	4	20	12.54	3.307
Valid N (list wise)	90				

When the results of the two approaches were compared, the digital method showed a statistically significant (P=0.007) greater increase in post-test scores (12.54-9.79 = 2.75) than the traditional way (11.24-10.43 = 0.81) (Table 4).

**Table 4:** Comparative analysis between digital and conventional methods

	Digital (n=90)	Conventional (n=90)	P-value
Pre-test	9.79	10.43	0.088
Post-test	12.54	11.24	0.007

**Figure 1:**
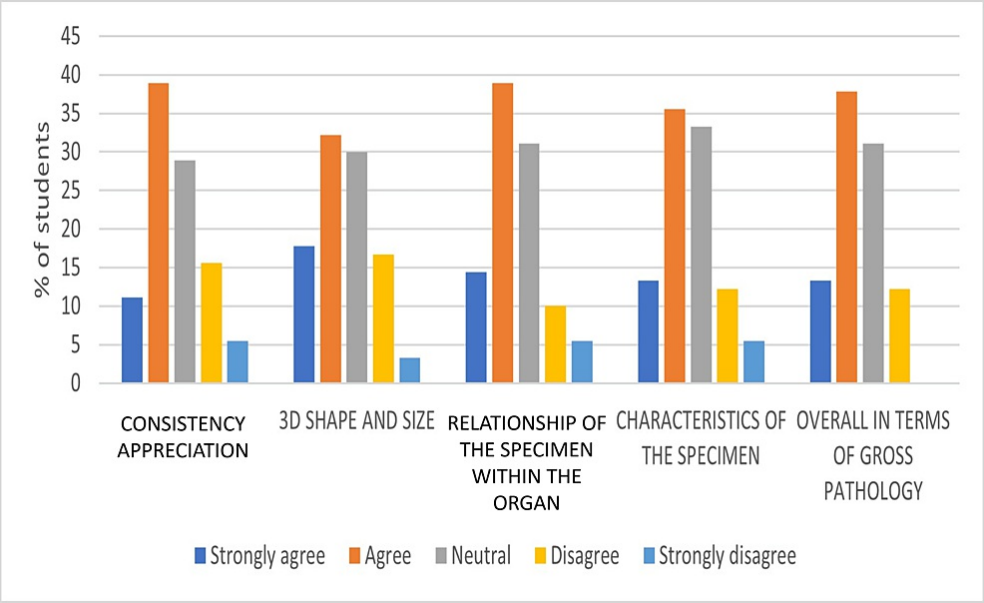
Student's responses on the superiority of the digital method in terms of gross pathology

Students who were asked about gross pathology preferred the digital technique in areas of consistency appreciation by 50%, 3D shape and size by 50%, relationship of the specimen within the organ by 53.5%, characteristics of the specimen by 48.9%, and overall pathology by 51.1% (strongly agree and agree), respectively. Less than 21% of students (strongly disagree and disagree) were not in favor of the digital method, while an average of 30.88% of students held neutral views (Figure 1).

**Figure 2:**
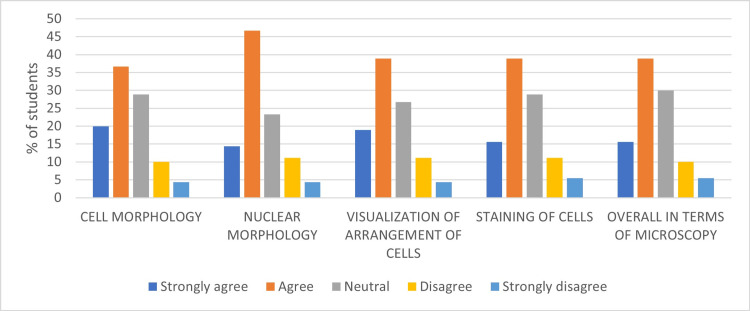
Student's responses on the superiority of the digital method in terms of microscopy

When students were asked their opinion on the superiority of the digital method in various aspects of microscopic pathology on a Likert’s scale, a total of 56.7% (strongly agree and agree) of students responded in favor of digital pathology concerning cell morphology, 61.1% of students for nuclear morphology, 57.8% of students concerning visualization of cell arrangement, 54.5% concerning staining of cells, and 54.5% of students concerning overall microscopic pathology. An average of 27.56% of students had a neutral opinion. No more than 15% (strongly disagree and disagree) of students in each category were not in favor of the digital method of microscopy (Figure 2).

## Discussion

In this study, we aimed to assess the influence of using virtual and conventional microscopy on students’ performance and whether their study behaviors were influenced by these tools.

In the teaching of general pathology and histology disciplines, several medical and dental schools have discussed their experiences with the switch from conventional to digital microscopy. In their study of switching from traditional glass slides to virtual microscopy in oral pathology lessons, Fonseca et al. noted noticeably higher ratings given to the effectiveness of the virtual microscopy in their questionnaire, which amply demonstrates the students' enthusiasm for and approval of this new methodological approach [13]. Similar findings can be seen in this study, where students favored the digital method as compared to a conventional method.

Under a light microscope, the field cannot be changed to guarantee that each student sees the same field. Thus, students are unable to view the entire slide as a result. At the same time, it is also impossible to see the intricate details of any tissue at a higher magnification by changing the objective. Also, the slides must be replaced frequently to prevent color fading or slide damage.

A study done by Hande et al. found that students frequently become confused when the slide collection's sections differ, which hinders the effectiveness of the teaching process. His study also showed better results with virtual microscopy, and at the same time, he emphasized the collaborative use of virtual and conventional microscopy to improve learning [12]. Light microscopy sections cannot be standardized, nor can the typical areas that the students need to visualize. When using a microscope to examine histopathological slides, medical students frequently lament their inability to detect lesions. The penta-head and deca-head microscopes are a partial solution, but they can also be used for a small group.

A study done by Hamilton describes that the cost of obtaining and maintaining equipment for teaching pathology through microscopy and gross specimens can be expensive, especially for rare diseases and educational institutions with limited resources. Digital pathological teaching methods can save time for both students and instructors. According to a study by Pantanowitz et al., digital pathology has the potential to reduce the time needed for the preparation and delivery of pathology teaching materials, thereby increasing efficiency and productivity [14,15]. Digital slides always have the same quality. They never break, get lost, or deteriorate with time. Thus, it is not necessary to replace them by recutting and staining new slides every certain period, with the subsequent impact on costs and preservation of the frequently highly valuable tissue from the paraffin block [15,16], and it can be sent between remote locations via telecommunications technology, making it possible to conduct research, diagnose, and educate using image-rich pathology data. Additionally, access to large digital archives of tissue slides could be an invaluable educational resource for students and residents in pathology [17].

Another study by Pantanowitz et al. evaluated the use of digital microscopy in teaching pathology to medical students in a resource-limited setting and found that digital microscopy was a cost-effective and efficient method of teaching pathology, particularly in settings where conventional microscopy is not feasible due to limited resources [18]. Overall, teaching pathology via microscopy and gross specimens has advantages and disadvantages. However, the benefits of accurate diagnosis and hands-on experience may outweigh the challenges of limited access to specimens, cost, and time. It is essential to weigh the pros and cons and choose the most appropriate teaching methods based on the educational institution's resources and goals. Our study had similar results, where the digital method had better results as compared to other methods of teaching surgical pathology.

The results thus highlight the fact that the use of virtual slides is now an essential teaching tool and reveal a noticeable improvement in the student's commitment to and excitement for studying pathology. Virtual platforms and software tools can allow for real-time communication and collaboration between students and instructors [11] by offering interactive learning tools such as virtual microscopy, animations, and videos, which help medical students understand pathology concepts better [19].

Many viewers enable the presentation of multiple slides at once, making it easier to comprehend immunohistochemical methods by contrasting them with the traditional hematoxylin-eosin stain [20,21]. Because students utilizing whole slides do not learn how to use the conventional microscope, there is still considerable debate regarding whether whole-slide imaging can completely replace it. The initial financial outlay for the purchase of the scanner and the entire slide imaging system is the biggest drawback of whole slide imaging [20,22].

The same level of hands-on experience and practical training as traditional classroom-based learning cannot be provided. This can be particularly important in the field of pathology, where hands-on experience is crucial for developing diagnostic skills [9,19].

Another study by Abdollahi et al. showed a similar mean score with conventional and digital teaching methods. Not many comparative studies are available, which is why more comparative research like this should be done with a larger sample and at various parts to have more credible data and reach a particular outcome [23].

The future of teaching pathology to medical students is likely to involve a combination of digital and conventional methods, with digital methods playing an increasingly important role. Virtual reality (VR) and augmented reality (AR) technologies are also being explored as potential tools for teaching pathology. These technologies allow students to interact with three-dimensional models of organs and tissues, providing a more immersive and engaging learning experience [24].

However, conventional methods of teaching pathology, such as gross specimen dissection and observation under a microscope, are still important for developing practical skills and understanding the physical properties of tissue samples. These methods can also be used in combination with digital tools to provide a more comprehensive learning experience [25].

Another potential of digital pathology is artificial intelligence (AI). It is an emerging technology in the field of science. In simple terms, it is programming a machine to solve problems without the need to specify a solution at each step to reach the result [26]. AI-equipped radiology machines are already in use and are constantly being tested to make improvements to aid the radiologist in making a definitive diagnosis. Similarly, AI nanobots are used for targeted therapy for cancer to target specific tumor cells in the body and avoid various side effects of chemotherapy and radiotherapy. AI in pathology will help pathologists cover many samples, thus helping in various screening programs to test a huge number of populations. It can also help pathologists by pointing out minor details that are sometimes missed due to operator-based dependency on the microscopes.

Overall, while there may be potential benefits to teaching pathology via microscopy and gross specimens, it is important to consider the use of technology and other innovative approaches to enhance the learning experience and improve diagnostic accuracy.

## Conclusions

Digital methods of teaching surgical pathology not only improved the performance of students but also led to a better understanding of the subject, as observed by the significant P-value. However, we do not recommend the complete replacement of conventional methods of teaching by digital methods; the use of digital methods as an additional mode of imparting practical training to students will improve their understanding of gross and microscopy surgical pathology.
